# Anxiety and depression in keratotic oral lichen planus: a multicentric study from the SIPMO

**DOI:** 10.1007/s00784-023-04909-3

**Published:** 2023-02-14

**Authors:** Daniela Adamo, Elena Calabria, Federica Canfora, Noemi Coppola, Stefania Leuci, Martina Mignogna, Lorenzo Lo Muzio, Francesca Spirito, Michele Giuliani, Lorenzo Azzi, Marta Dani, Giuseppe Colella, Chiara Colella, Lucio Montebugnoli, Davide Bartolomeo Gissi, Mario Gabriele, Marco Nisi, Andrea Sardella, Giovanni Lodi, Elena Maria Varoni, Amerigo Giudice, Alessandro Antonelli, Alessio Gambino, Giuliana Antonucci, Paolo Vescovi, Marco Meleti, Alessandra Majorana, Elena Bardellini, Giuseppina Campisi, Vera Panzarella, Francesco Spadari, Umberto Garagiola, Monica Pentenero, Samuele Sutera, Matteo Biasotto, Giulia Ottaviani, Margherita Gobbo, Luca Guarda Nardini, Umberto Romeo, Gianluca Tenore, Rosario Serpico, Alberta Lucchese, Carlo Lajolo, Gioele Gioco, Massimo Aria, Luca D’Aniello, Michele Davide Mignogna, SIPMO (Italian Society of Oral Pathology, Medicine)

**Affiliations:** 1 Department of Neuroscience, Reproductive Sciences and Dentistry, University of Naples Federico II, Naples, Italy; 2 Department of Clinical and Experimental Medicine, University of Foggia, Foggia, Italy; 3 Unit of Oral Medicine and Pathology, ASST Dei Sette Laghi, Department of Medicine and Surgery, University of Insubria, Varese, Italy; 4 Multidisciplinary Department of Medical, Surgical and Dental Specialties, University of Campania Luigi Vanvitelli, Naples, Italy; 5 Department of Biomedical and Neuromotor Sciences, Section of Oral Sciences, University of Bologna, Bologna, Italy; 6 Department of Surgical Pathology, Medicine, Molecular and Critical Area, University of Pisa, Pisa, Italy; 7 Department of Biomedical, Surgical and Dental Sciences, University of Milan, Milan, Italy; 8 Department of Health Sciences, Magna Graecia University of Catanzaro, Catanzaro, Italy; 9 Oral Medicine Section, Department of Surgical Science, CIR Dental School, University of Turin, Turin, Italy; 10 Department of Medicine and Surgery, Oral Medicine and Laser Surgery Unit, University Center of Dentistry, University of Parma, Parma, Italy; 11 Department of Medical and Surgical Specialities, Radiological Sciences and Public Health, University of Brescia, Brescia, Italy; 12 Department of Surgical, Oncological, and Oral Sciences, University of Palermo, Palermo, Italy; 13 Department of Biomedical, Surgical and Dental Sciences, Maxillo-Facial and Dental Unit, Fondazione IRCCS Ca’ Granda Ospedale Maggiore Policlinico, University of Milan, Milan, Italy; 14 Department of Oncology, Oral Medicine and Oral Oncology Unit, University of Turin, Turin, Italy; 15 Department of Medical, Surgical and Health Sciences, University of Trieste, Trieste, Italy; 16 Unit of Oral and Maxillofacial Surgery, Ca’ Foncello Hospital, Treviso, Italy; 17 Department of Oral Sciences and Maxillofacial Surgery, University of Rome La Sapienza, Rome, Italy; 18 Head and Neck Department, Fondazione Policlinico Universitario A. Gemelli IRCCS, School of Dentistry, Università Cattolica del Sacro Cuore, Rome, Italy; 19 Department of Economics and Statistics, University Federico II of Naples, Naples, Italy; 20 Department of Social Sciences, University Federico II of Naples, Naples, Italy

**Keywords:** Keratotic oral lichen planus, Depression, Anxiety, Mood disorder, Pain

## Abstract

**Objectives:**

Oral lichen planus with exclusive keratotic reticular, papular, and/or plaque-like lesions (K-OLP) is a clinical pattern of OLP that may be associated with a complex symptomatology and psychological alteration. The aim of the study was to evaluate the prevalence of anxiety (A) and depression (D) in patients with K-OLP, analyzing the potential predictors which can affect mental health status.

**Methods:**

Three hundred K-OLP patients versus 300 healthy controls (HC) were recruited in 15 Italian universities. The Numeric Rating Scale (NRS), Total Pain Rating Index (T-PRI), and Hamilton Rating Scales for Depression and for Anxiety (HAM-D and HAM-A) were administered.

**Results:**

The K-OLP patients showed statistically higher scores in the NRS, T-PRI, HAM-D, and HAM-A compared with the HC (*p*-value < 0.001^**^). A and D were found in 158 (52.7%) and 148 (49.3%) K-OLP patients. Strong linear correlations were identified between HAM-A, HAM-D, NRS, T-PRI, and employment status and between HAM-D, HAM-A, NRS, T-PRI, employment status, and female gender. Multivariate logistic regression revealed that HAM-D and HAM-A showed the greatest increase in the R2 value for A and D in the K-OLP patients, respectively (DR2 = 55.5% *p*-value < 0.001**; DR2 = 56.5% *p*-value < 0.001**).

**Conclusions:**

The prevalence of A and D is higher in the K-OLP patients compared with the HC, also found in K-OLP subjects without pain, suggesting that the processing of pain may be in a certain way independent of the processing of mood.

**Clinical relevance:**

Mood disorders and pain assessment should be carefully performed in relation to K-OLP to obtain a complete analysis of the patients.

**Supplementary Information:**

The online version contains supplementary material available at 10.1007/s00784-023-04909-3.

## Introduction

Oral lichen planus (OLP) is a chronic immune-mediated, inflammatory disease of the oral mucosa, affecting 1.01% of the population with a higher prevalence in Europe (1.38%) [1, 2]. The pathogenic mechanism of the illness remains unknown but genetic, environmental, and local factors and psychological distress may have a role in the activation of the host immunological system against the oral mucosa, supporting the hypothesis of an immune-mediated disease [3, 4].

OLP may present with different clinical patterns, ranging from keratotic manifestations (white reticular, papular, and/or plaque-like lesions), usually symmetrical and bilateral, to predominantly non-keratotic OLP (atrophic, erythematous, erosive, ulcerative and/or bullous lesions) [2, 4]. Moreover, OLP is included among the group of potentially malignant disorders of the oral mucosa, with a risk of progression to cancer of 2.28% [5].

Despite the OLP with exclusive keratotic manifestations (K-OLP) being usually considered asymptomatic compared with non-keratotic OLP, recent studies have suggested a high prevalence of oral discomforts, a burning sensation and pain with additional oral symptoms such as taste disturbance, xerostomia, and globus pharyngeus (a non-painful sensation of a lump or foreign body in the throat). Such symptoms are not related to clinical features because they have been revealed also in oral sites without lesions [6–8].

The oral symptomatology associated with the fear of cancer development may contribute to emotional and mood changes [9, 10], as suggested by the higher levels of anxiety (A) and depression (D) reported in these patients compared with healthy subjects. In addition, several studies have highlighted that A and D may be considered as triggers both in relation to the onset but also to the exacerbation of the disease, which in turn may amplify the subjective oral symptoms [11–13]. The synergic association between OLP, A, and D may further contribute to a poor quality of life and an increased level of stress among such subjects. [14]

Despite recent studies have suggested a strong association between OLP and mood disorders [7, 15, 16], with an overall estimated prevalence of 54.76% and 31.19% of OLP patients suffering from A and D [1], few studies have analyzed the prevalence of A and D in a subset of patients with K-OLP. Therefore, we decided to perform a multicentric study in our country in a large cohort of patients with K-OLP to confirm this association and to better understand the role of the sociodemographic profile, risk factors, and oral symptoms in the development of A and D in this subgroup of OLP patients. Thus, the aim of this study was to evaluate the prevalence of A, D, pain, and additional intra-oral symptoms in a wide cohort of Italian patients with K-OLP, compared with a group of healthy controls (HC), analyzing the predictors that can cause this psychological impairment.

## Materials and methods

This study is a descriptive secondary analysis of a multicentric clinical observational study which was conducted between January 2019 and February 2020, involving fifteen Italian University departments of Oral Medicine belonging to the Italian Society of Oral Pathology and Medicine (Società Italiana di Patologia e Medicina Orale) (SIPMO) [6].

The study was previously approved by the Ethics Committee of Federico II University of Naples, (Approval Number:184/18) and conducted in accordance with the ethical principles of the World Medical Association Declaration of Helsinki. The adopted methods conformed with the Strengthening of the Reporting of Observational Studies in Epidemiology (STROBE) guidelines for observational studies [17].

### Participants and procedure

As described in our previous research, [6] all potentially eligible participants were invited to participate in the study and provided their written informed consent. The inclusion and exclusion criteria were established in accordance with the previous SIPMO studies, in order to follow the same guidelines.

Additionally, the procedures used to enlist the patients in the group and the screening process conformed with those adopted in the other studies, involving expert clinicians in oral medicine and psychiatry for the psychological assessment. Sociodemographic characteristics were formerly recorded, such as the anamnestic evaluation [6]. The acronym K-OLP (keratotic oral lichen planus) refers to the OLP characterized by the presence of exclusive keratotic manifestations, namely papular, reticular, and/or plaque-like white lesions with histopathology showing the presence of keratosis. As previously described, in all cases the OLP diagnosis was based on clinical and histopathological findings [6].

Questionnaires were administered to the K-OLP patients and HC in order to completely analyze the intensity of pain, through the Numerical Rating Scale (NRS) [18, 19]; the quality of pain experienced, through the Total Pain Rating Index (T-PRI) [20]; D, using the Hamilton Rating Scale for Depression (HAM-D) [21, 22], and A, with the Hamilton Rating Scale for Anxiety (HAM-A) [23]. These questionnaires have been described in detail in our previous studies [6, 7].

### Statistical analysis

The statistical analysis was performed using the SPSS software v. 23. Descriptive statistics, including means, standard deviations, medians, and interquartile range (IQR), were used to analyze the socio-demographic and clinical characteristics of the groups. The Pearson chi-square test and Fisher’s exact test were used to assess the significant differences between the percentages, and the differences associated with *p*-values less than 0.05 or 0.01 were considered moderately or strongly significant, respectively. The non‐parametric Mann‐Whitney U test was employed to evaluate differences between the median scores of the HAM-A, HAM-D, NRS, and T-PRI in the groups. P‐values < 0.05 were considered to reflect a statistical significance. The Spearman test was used to analyze the correlation between the qualitative and quantitative predictors and HAM-A and HAM-D median scores.

To identify potential predictors of A and D in K-OLP, multiple linear regression analyses were performed, considering sociodemographic parameters (age, gender, education, marital status, employment status) and intensity and quality of pain (NRS and T-PRI). Full models, when all the parameters were entered simultaneously, were used to evaluate the relative contributions of these variables to pain.

In detail, a sequential regression model analysis including predictors, one by one, to obtain unadjusted coefficient estimations was performed. Moreover, in a final step, we performed a full model analysis considering all predictors, simultaneously, to estimate adjusted coefficients. In all steps, we provided standard errors of model coefficients which measure the statistical precision of inference estimation of the model parameters.

### Sample size calculation

The sample size, namely 300 subjects, was set by fixing a test power of no less than 90% associated with a significance of no more than 5% (software G*Power 3.1.9.7 by Dusseldorf University). This sample size calculation was performed using the effect size estimation from a previously published research study regarding scales of mood disorders and pain [7, 24].

## Results

A total of 600 participants were enrolled, 300 K-OLP patients and 300 HC. The sociodemographic characteristics of both groups are shown in Table 1. In the sample of K-OLP and HC, the prevalence of women and men was 58.3% (175) and 41.7% (125), respectively, with no difference in the mean age between the two groups (*p*-value: 0.597). Statistically significant differences were found in relation to employment and marital status. A lower proportion of K-OLP patients were employed (108; 36%) compared to the HC (155; 51.7%) (*p*-value: 0.001**). In contrast, a statistically significant higher percentage of K-OLP patients were married (217; 72.3%) in comparison to the HC (176; 58.7%) (*p*-value: < 0.001**). In addition, the HC presented a higher education (*p*-value: < 0.001**) and were characterized by a significantly higher percentage of smokers (32.0%) (*p*-value: 0.001**). No differences were detected in terms of BMI and alcohol consumption between the groups (*p*-values: 0.065, 0.767).

**Table 1:** Socio-demographic profile, body mass index, disease onset, and risk factors in the 300 K-OLP patients and 300 HC

Demographic variables	K-OLP patients	HC	*p*-value
	N° (%)	N° (%)	
Gender			
Male	125 (41.7)	125 (41.7)	1.000
Female	175 (58.3)	175 (58.3)	
Employment			
Employed	108 (36.0)	155 (51.7)	< 0.001**
Not employed	192 (64.0)	145 (48.3)	
Family situation			
Married	217 (72.3)	176 (58.7)	< 0.001**
Not married	83 (27.7)	124 (41.3)	
	Mean ± SD	Mean ± SD	*p*-value
Age (in years)	65.2 ± 12.2	64.2 ± 16.9	0.597
Education (in years)	10.9 ± 4.05	13.6 ± 4.5	< 0.001**
Body mass index	24.9 ± 3.92	24.3 ± 3.63	0.065
Disease onset (years)	4.5 ± 2.3	/	/
Risk factors	**N° (%)**	**N° (%)**	*p*-value
Smoker			
Yes	66 (22.0)	96 (32.0)	0.001**
No	234 (78.0)	204 (68.0)	
Alcohol use			
Yes (≤ 14 units/week)	91 (30.3)	95 (31.7)	0.767
No	209 (69.7)	205 (68.3)	

The significant difference between the percentages was measured by the Pearson chi-square test. * Significant 0.01 < *p* ≤ 0.05, ** Significant *p* ≤ 0.01

Abbreviation: *K-OLP*, keratotic oral lichen planus; *HC*, healthy controls

Table 2 shows the distributions of systemic comorbidities and drug consumption among the groups. Specifically, a higher percentage of the K-OLP patients suffered from gastro-esophageal reflux (46; 15.3%) and from benign prostatic hypertrophy (21; 7.0%) compared to the HC (27; 9.0% and 8; 2.7% respectively) (*p*-values: 0.024* and 0.021*). Further, a moderately significant difference was observed with respect to the intake of levothyroxine sodium, which was more prevalent among the K-OLP group (36, 12%; *p*-value:0.023*). No further differences were recorded for all the other comorbidities and drugs.

**Table 2:** Frequency of systemic diseases and drug consumption in the 300 K-OLP patients and 300 HC

	K-OLP N° (%)	HC N° (%)	*P*-value
Systemic diseases			
Essential hypertension	98 (32.7)	78 (26.0)	0.088
Hypercholesterolemia	67 (22.3)	50 (16.7)	0.099
Gastro-esophageal reflux disease	46 (15.3)	27 (9.0)	0.024*
Hypothyroidism	34 (11.3)	21 (7.0)	0.089
Diabetes	26 (8.3)	21 (7.0)	0.598
Previous malignant disease	24 (8.0)	16 (5.3)	0.252
Benign prostatic hypertrophy	21 (7.0)	8 (2.75)	0.021*
Endocrine disease	11 (3.7)	6 (2.0)	0.325
Hepatitis C	10 (3.3)	4 (1.3)	0.174
Asthma	7 (2.3)	7 (2.3)	1.000
Previous myocardial infarction	6 (2.0)	8 (2.7)	0.788
Hyperthyroidism	5 (1.7)	4 (1.3)	1.000
Hepatitis B	4 (1.3)	0 (0.0)	0.124
Drug consumption			
Beta‐adrenergic receptor blockers	47 (15.7)	35 (11.7)	0191
Simvastatin	43 (14.3)	41 (13.7)	0.906
Proton pump inhibitors	42 (14.0)	35 (11.7)	0.464
Levothyroxine sodium	36 (12)	19 (6.3)	0.023*
Antiplatelets	35 (11.7)	24 (8.0)	0.17
ACE-inhibitors	28 (9.3)	31 (10.3)	0.784
Angiotensin II receptor blockers	24 (8.0)	17 (5.7)	0.332
Diuretics	24 (8.0)	24 (8.0)	1.000
Metformin	24 (8.0)	16 (5.3)	0.252
Blood thinner	15 (5.0)	6 (2.0)	0.073
Insulin	8 (2.7)	6 (2.0)	0.788

The significant difference between percentages was measured by Fisher’s exact test. * Significant 0.01 < *p* ≤ 0.05, ** Significant *p* ≤ 0.01

Abbreviation: *K-OLP*, keratotic oral lichen planus; *HC*, healthy controls

Table 3 shows the frequencies of the oral symptoms for the K-OLP and the HC groups. The most frequently reported oral symptom among the K-OLP group was a painful sensation (149; 58.3%), described as burning in character, which was localized in one or more sites in 43.0% (103) of the patients while in 15.3% (46) it was diffuse throughout the oral mucosa even where there were no lesions [6]. A statistically strongly significant higher percentage of the K-OLP patients suffered from pain/burning, xerostomia, dysgeusia, subjective halitosis, globus pharyngeus, itching, intraoral foreign body sensation, tingling sensation (*p*-value: < 0.001**) and dysosmia (*p*-value: 0.002**) in comparison with the HC. A moderately significant difference was found with respect to sialorrhea (*p*-value: 0.032*), and occlusal dysesthesia (*p*-value: 0.03*) while no difference was revealed with regard to oral dyskinesia (*p*-value: 0.068) and a change in the tongue morphology (*p*-value: 1.000).

**Table 3:** Frequency of oral symptoms in the 300 K-OLP patients and 300 HC

Oral symptoms	K-OLP N° (%)	HC N° (%)	*P*-value
Pain/burning localized in one or more sites	103 (43.0)	15 (5.4)	< 0.001**
Pain/burning diffuse	46 (15.3)	7 (2.3)	< 0.001**
Xerostomia	101 (33.7)	33 (11)	< 0.001**
Dysgeusia	58 (19.3)	13 (4.3)	< 0.001**
Subjective halitosis	55 (18.3)	27 (9)	0.001**
Globus pharyngeus	40 (13.3)	11 (3.7)	< 0.001**
Intraoral foreign body sensation	35 (11.7)	10 (3.3)	< 0.001**
Itching	34 (11.4)	9 (3)	< 0.001**
Sialorrhea	31 (10.3)	16 (5.3)	0.032*
Tingling sensation	29 (9.7)	6 (2)	< 0.001**
Occlusal dysesthesia	23 (7.7)	10 (3.3)	0.03*
Dysosmia	19 (6.3)	4 (1.3)	0.002**
Oral dyskinesia	7 (2.3)	1 (0.3)	0.068
Change in tongue morphology	2 (0.7)	1 (0.3)	1.000

The significant difference between the percentages was measured by Fisher’s exact test. * Significant 0.01 < *p* ≤ 0.05, ** Significant *p* ≤ 0.01

Abbreviation: *K-OLP*, keratotic oral lichen planus; *HC*, healthy controls

Table 4 shows the median and interquartile range of the clinical parameters (NRS, T-PRI, HAD-A, and HAD-D) and a comparison of the frequencies of the patients and controls in relation to the severity of the pain and the psychological parameters.

**Table 4:** Score analysis of the pain, anxiety, and depression of the 300 K-OLP patients and 300 HC

	K-OLP	HC	*P*-value
Clinical parameters	Median; IQR	Median; IQR	
NRS	2 [0–5]	0 [0–0]	< 0.001**
T-PRI	2 [0–5]	0 [0–0]	< 0.001**
HAM-A	7 [3–12]	5 [1–10]	< 0.001**
HAM-D	6 [3-12]	5 [2–9]	< 0.001**
Score analysis of pain intensity	N° (%)	N° (%)	*p*-value
NRS			
Absent (0)	119 (39.7)	242 (80.7)	< 0.001**
Mild pain (1–4)	91 (30.3)	36 (12)	
Moderate pain (5–6)	51 (17)	14 (4.7)	
Severe pain (7–10)	39 (13)	8 (2.7)	
Score analysis of psychological parameters	**N° (%)**	N° (%)	*p*-value
HAM-A			
No anxiety (< 7)	142 (47.3)	186 (62)	< 0.001**
Mild (7–17)	122 (40.7)	96 (32)	
Moderate (18–24)	23 (7.7)	15 (5)	
Severe (> 24)	13 (4.3)	3 (1)	
HAM-D			
No depression (< 7)	152 (50.7)	192 (64)	0.003**
Mild (7–17)	118 (39.3)	94 (31)	
Moderate (18–24)	23 (7.7)	9 (3)	
Severe (> 24)	7 (2.3)	5 (1.7)	

IQR is the interquartile range. The significant difference between medians was measured by the Mann–Whitney *U* test

^*^ Significant 0.01 < *p* ≤ 0.05, ** Significant *p* ≤ 0.01

The significant difference between the HAM-A and HAM-D percentages affected by HAM-A and HAM-D was measured by Fisher’s exact test. * Significant 0.01 < *p* ≤ 0.05, ** Significant *p* ≤ 0.01

Abbreviations: *K-OLP*, keratotic oral lichen planus; *HAM-A*, Hamilton Rating Scale for Anxiety; *HAM-D*, Hamilton Rating Scale for Depression; *HC*, healthy controls; *NRS*, Numeric Rating Scale; *T-PRI*, Total Pain Rating Index

Overall, the K-OLP patients presented statistically significantly higher median total scores for the NRS, T-PRI, HAM-A, and HAM-D in comparison to the HC (*p*-values: < 0.001**). Additionally, there was a significantly different frequency distribution between the K-OLP patients and the HC with respect to the NRS categories (*p*-values: < 0.001**), as only 39.7% (119) of the K-OLP group presented no pain compared to 80.7% (242) of the HC. In detail, 30.3% (91), 17% (51), and 13% (39) of the K-OLP patients presented mild, moderate, and severe pain, respectively, compared to 12% (36), 4.7% (14), and 2.7% (8) of the HC. Statistically significant differences in the frequency distributions were also detected when analyzing the severity scores of the HAM-A and HAM-D (*p*-values: < 0.001** and 0.003** respectively). Indeed, no A was reported in 47.3% (142) of the K-OLP patients compared to 62% (186) of the HC. A was found in 52.7% (158) of the K-OLP group; 40.7% (122) presented mild A, 7.7% (23) moderate A, and 4.3% (13) severe A in comparison with 32% (96), 5% (15) and 1% (3) of the HC, respectively. With regard to the HAM-D score categories, instead, 50.7% (152) of the K-OLP patients reported no D compared with 64% (192) of the HC. D was found in 49.3% (148) of the K-OLP group; 39.3% (118) presented mild D, 7.7% (23) moderate D, and 2.3% (7) severe D, compared with 31% (94), 3% (9), and 1.7% (5) of the HC.

Table 5 shows the frequency distributions of A and D (the HAM-A and HAM-D scores) in relation to the intensity of pain (the NRS scores). Interestingly, A and D were reported in 43.7% (52) and in 47% (56) of the patients with K-OLP without pain. In particular, we have also found that the 62.3% (109) of K-OLP females suffered from A versus the 38.4% (48) of K-OLP males and that the 57.1% (100) of K-OLP females suffered from depression versus the 37.6% (47) of K-OLP males (data not displayed in the table).

**Table 5:** Frequency distribution of anxiety and depression scores (HAM-A, HAM-D) depending on the severity of the pain (NRS)

Score analysis of psychological parameters versus pain intensity	K-OLP N° (%)				*P*-value	HC N° (%)				*P*-value
	NRS					NRS				
	*Absent*	*Mild pain*	*Moderate pain*	*Severe pain*		*Absent*	*Mild pain*	*Moderate pain*	*Severe pain*	
HAM-A										
No anxiety (< 7)	67 (56.3)	45 (49.5)	21 (41.2)	9 (23.1)	0.003**	158 (65.3)	22 (61.1)	3 (21.4)	3 (37.5)	0.001**
Mild (7–17)	46 (38.7)	37 (40.7)	19 (37.3)	20 (51.3)		75 (31)	10 (27.8)	8 (57.1)	3 (37.5)	
Moderate (18–24)	3 (2.5)	5 (5.5)	8 (15.7)	7 (17.9)		7 (2.9)	4 (11.1)	2 (14.3)	2 (25)	
Severe (> 24)	3 (2.5)	4 (4.4)	3 (5.9)	3 (7.7)		2 (0.8)	0 (0)	1 (7.1)	0 (0)	
HAM-D										
No depression (< 7)	63 (52.9)	52 (57.1)	22 (43.1)	15 (38.5)	0.093	160 (66.1)	23 (63.9)	5 (35.7)	4 (50)	0.012*
Mild (7–17)	47 (39.5)	34 (37.4)	21 (41.2)	16 (41)		74 (30.6)	9 (25)	8 (57.1)	3 (37.5)	
Moderate (18–24)	8 (6.7)	3 (3.3)	7 (13.7)	5 (12.8)		7 (2.9)	2 (5.6)	0 (0)	0 (0)	
Severe (> 24)	1 (0.8)	2 (2.2)	1 (2)	3 (7.7)		1 (0.4)	2 (5.6)	1 (7.1)	1 (12.5)	

The significance difference between the NRS percentages affected by HAM-A and HAM-D among the K-OLP patients and controls was measured by the Fisher’s exact test. * Significant 0.01 < *p* ≤ 0.05, ** Significant *p* ≤ 0.01

Abbreviations: K-OLP, keratotic oral lichen planus; HAM-A, Hamilton Rating Scale for Anxiety; HAM-D, Hamilton Rating Scale for Depression; HC, healthy controls; NRS, Numeric Rating Scale

Tables 6 and 7 show the dependence analyses between the HAM-A and HAM-D scores and the quantitative and qualitative variables. There was a positive correlation between the HAM-A scores and the HAM-D, NRS, and T-PRI scores (*p*-values: < 0.001**), while no correlation was found with age, education, and BMI (*p*-values: 0.132, 0.051, 0.553 respectively). Similarly, a positive correlation was also found between the HAM-D scores and the HAM-A, NRS, and T-PRI scores (*p*-values: < 0.001**). Furthermore, both the HAM-A and HAM-D scores were positively correlated with the female gender and with unemployment status (HAM-A *p*-values: 0.001**, 0.022*; HAM-D *p*-values: 0.010**, 0.020*).

**Table 6:** Dependence analysis between HAM-A and quantitative and qualitative predictors in the K-OLP patients

	HAM-A	
Quantitative predictors	*p*-value	
Age	0.087 (0.132)	
Education	− 0.116 (0.051)	
BMI	0.034 (0.553)	
HAM-D	0.742 (< 0.001**)	
NRS	0.231 (< 0.001**)	
T-PRI	0.333 (< 0.001**)	
Qualitative predictors	*Median [Q1:Q3]*	*p*-value
Gender		
Female	8 [4–13]	0.001**
Male	5 [1.5–11]	
Marital status		
Married	7 [3–11.2]	0.537
Not married	7.5 [3–12.5]	
Employment status		
Employed	6 [1–12]	0.022*
Not employed	8 [4–12]	
Smoking status		
Smoker	9 [4–15]	0.059
Non-smoker	7 [3–11]	
Alcohol use		
Yes	6 [2–12]	0.126
No	8 [4–11.8]	

*r* is Spearman’s correlation coefficient. *p*-value—*Significant 0.01 < *p*-value ≤ 0.05. **Significant *p*-value ≤ 0.01

The significant difference between the medians was measured by the Mann–Whitney *U* test

Abbreviations: *BMI*, body mass index; *K-OLP*, keratotic oral lichen planus; *HAM-A*, Hamilton Rating Scale for Anxiety; *HAM-D*, Hamilton Rating Scale for Depression; *HC*, healthy controls; *NRS*, Numeric Rating Scale; *T-PRI*, Total Pain Rating Index

**Table 7:** Dependence analysis between HAM-D and quantitative and qualitative predictors in the K-OLP patients

	HAM-D	
Quantitative predictors	*p*-value	
Age	0.096 (0.095)	
Education	− 0.089 (0.136)	
BMI	0.029 (0.613)	
HAM-A	0.742 (< 0.001**)	
NRS	0.153 (< 0.001**)	
T-PRI	0.273 (< 0.001**)	
Qualitative predictors	*Median [Q1:Q3]*	*p*-value
Gender		
Female	7 [3–13]	0.010**
Male	5 [2–9.5]	
Marital status		
Married	6 [3–11]	0.145
Not married	8.5 [2.75–14.2]	
Employment status		
Employed	5 [1.75–9.25]	0.020*
Not employed	7 [3–13]	
Smoking status		
Smoker	8.5 [3–13.2]	0.219
Non-smoker	6 [3–11]	
Alcohol use		
Yes	6 [2–10]	0.204
No	7 [3.25–12]	

*r* is Spearman’s correlation coefficient. *p*-value—*Significant 0.01 < *p*-value ≤ 0.05. **Significant *p*-value ≤ 0.01

The significant difference between the medians was measured by the Mann–Whitney *U* test

Abbreviations: *BMI*, body mass index; *K-OLP*, keratotic oral lichen planus; *HAM-A*, Hamilton Rating Scale for Anxiety; *HAM-D*, Hamilton Rating Scale for Depression; *HC*, healthy controls; *NRS*, Numeric Rating Scale; *T-PRI*, Total Pain Rating Index

The results of the simultaneous multiple linear regression analyses for the K-OLP group, predicting A and D (HAM-A and HAM-D) are shown in Table 8. The first model tests the contribution of the demographic variables to A (HAM-A) revealing that only the female gender was found to be statistically moderately significant (*p*-value 0.046*). Instead, the addition of D (model 2) resulted in a significant increase in the R2 value for HAM-A (DR2 = 55.5%; *p*-value < 0.001**), similar to the addition of pain intensity (NRS) (model 3) (DR2 = 6.2%; *p*-value < 0.001**) and the addition of pain quality (T-PRI) (model 4) (DR2 = 20%; *p*-value < 0.001**). The final full model (model 5) in which all of the variables were entered simultaneously could explain 59.8% of the variance in the total scores of the HAM-A for the K-OLP patients (*p*-value: 0.001**). With respect to D (HAM-D), no demographic variables (model 1) were found to be statistically significant in the increase in the R2 value, while the addition of A (HAM-A) contributed to a significant increase in the R2 value (DR2 = 56.2%, *p*-value: < 0.001**). The addition of pain intensity (NRS) and pain quality (T-PRI) in models 3 and 4, respectively, resulted in an increase in the R2 value for the HAM-D scores (NRS DR2 = 3.6%, *p*-value: < 0.001**; T-PRI DR2 = 16.9%, *p*-value: < 0.001**). The final full model (model 5) could explain 58.7% of the variance of the HAM-D total scores for the K-OLP patients. (*p*-value: 0.001**).

**Table 8:** Multiple linear regression analysis predicting HAM-A and HAM-D in the 300 K-OLP patients

	Model 1		Model 2		Model 3		Model 4		Model 5	
	Beta (SE)	*P*-value	Beta (SE)	*P*-value	Beta (SE)	*P*-value	Beta (SE)	*P*-value	Beta (SE)	*P*-value
HAM-A K-OLP										
Age	− 0.01 (0.05)	0.860	0.01 (0.03)	0.729	− 0.01 (0.05)	0.869	− 0.03 (0.04)	0.521	0.01 (0.03)	0.755
Gender (F)	1.99 (0.99)	0.046*	0.95 (0.66)	0.153	1.19 (0.98)	0.227	0.78 (0.89)	0.387	0.36 (0.64)	0.574
Years of education	− 0.19 (0.13)	0.144	− 0.13 (0.09)	0.134	− 0.18 (0.13)	0.152	− 0.15 (0.12)	0.184	− 0.11 (0.08)	0.176
Marital status (married)	− 1.28 (1.07)	0.233	− 0.36 (0.71)	0.608	− 1.13 (1.03)	0.275	− 0.8 (0.95)	0.401	− 0.33 (0.67)	0.625
Employment status (employed)	− 0.04 (1.22)	0.973	0.66 (0.81)	0.415	− 0.33 (1.18)	0.779	− 0.92 (1.09)	0.402	0.54 (0.78)	0.487
HAM-D			0.82 (0.04)	< 0.001**					0.65 (0.05)	< 0.001**
NRS					0.69 (0.16)	< 0.001**			− 0.01 (0.14)	0.927
T-PRI							0.76 (0.09)	< 0.001**	0.25 (0.09)	0.006**
* R*^*2*^ (%)	1.6	0.093	57.1	< 0.001**	7.8	< 0.001**	21.6	< 0.001**	61.4	< 0.001**
* R*^*2*^*change* (%)			55.5	< 0.001**	6.2	< 0.001**	20	< 0.001**	59.8	< 0.001**
HAM-D K-OLP										
Age	− 0.02 (0.05)	0.596	− 0.02 (0.03)	0.542	− 0.03 (0.04)	0.594	− 0.04 (0.04)	0.324	− 0.03 (0.03)	0.329
Gender (F)	1.28 (0.92)	0.162	− 0.09 (0.61)	0.876	0.71 (0.92)	0.441	0.26 (0.85)	0.757	− 0.44 (0.61)	0.469
Years of education	− 0.08 (0.12)	0.531	0.06 (0.08)	0.476	− 0.07 (0.12)	0.562	− 0.05 (0.11)	0.681	0.06 (0.08)	0.471
Marital status (married)	− 1.12 (0.98)	0.256	− 0.23 (0.65)	0.719	− 1.01 (0.96)	0.294	− 0.72 (0.89)	0.423	− 0.22 (0.63)	0.729
Employment status (employed)	− 0.86 (1.12)	0.446	− 0.83 (0.74)	0.265	− 1.07 (1.10)	0.336	− 1.59 (1.03)	0.122	− 0.81 (0.73)	0.267
HAM-A			0.69 (0.04)	< 0.001**					0.58 (0.05)	< 0.001**
NRS					0.49 (0.15)	< 0.001**			− 0.25 (0.13)	0.050*
T-PRI							0.64 (0.09)	< 0.001**	0.22 (0.09)	0.014*
* R*^*2*^ (%)	0.3	0.333	56.5	< 0.001**	3.9	0.009**	17.2	< 0.001**	59.0	< 0.001**
* R*^*2*^*change* (%)			56.2	< 0.001**	3.6	< 0.001**	16.9	< 0.001**	58.7	< 0.001**

SE are the standard errors of the beta estimates. The *p*-values were obtained from the hypothesis test on the regression coefficients. *Moderately significant .01 < *p*-value ≤ .05.**Strongly significant *p*-value ≤ .01

Abbreviations: *K-OLP*, keratotic oral lichen planus; *HAM-A*, Hamilton Rating Scale for Anxiety; *HAM-D,* Hamilton Rating Scale for Depression; *HC*, healthy controls; *NRS*, Numeric Rating Scale; *T-PRI*, Total Pain Rating Index

## Discussion

The bidirectional link between mood disorders and OLP is well recognized in the literature in that patients with A and D had an almost three or four times greater risk of developing OLP compared with subjects without any psychological impairment [24, 25]. On the other hand, OLP patients are more prone to develop psychiatric comorbidities [16, 26].

A and D are the most common medical comorbidities associated with OLP, as suggested by several studies [27, 28] and by a recent systematic review and meta-analysis [1], especially in patients with non-keratotic OLP, a condition which exhibits symptomatic lesions and a higher level of intensity and quality of pain [6, 29]. However, the high prevalence of psychological distress and an unexpected symptomatology has been found also in the subset of patients with K-OLP [6, 7, 24, 30], a finding which continues to be an enigma considering that this subtype is considered to be asymptomatic and with a lower risk of cancerization, with the result that patients are not often followed-up in most countries [4, 5, 31].

The results of this study showed a higher prevalence of A and D in patients with K-OLP compared with HC. Indeed, A and D, respectively, were found in 52.7% (158) and 49.3% (148) of K-OLP patients, with the majority showing mild A (122; 40.7%) and mild D (118; 39.3%). The prevalence of A in K-OLP found in this study is in line with the data of a recent systematic review and meta-analysis of De Porras-Carrique T et al. [1], which has included fifty-one studies (with a total of 6815 patients). However, no data have been reported about differences between the subtypes of OLP, while, instead, the prevalence of D is higher compared with this study (31.19%). This high prevalence of A and D in K-OLP patients is surprising considering that patients with a history or occurrence of psychiatric illness were excluded from the study. Consequently, the majority of the K-OLP patients were unaware that they were suffering from A and D and had never been evaluated by a psychiatrist. This finding may suggest that people continue to be reticent to reveal or recognize that they have any psychiatric disease, particularly in certain countries such as Italy.

Burning sensation was reported by 58.3% (149) of K-OLP patients and in 15.3% (46) of these cases, this was diffused to the whole oral mucosa, reported also in sites without any lesions [6]. Despite the fact that in the dependence analysis, the intensity and quality of pain was found to be correlated with A and D (*p*-value < 0.001**), from the analysis of multiple linear regression, NRS and T-PRI could explain only 6.2% and 20% and 3.9% and 17.2% of the variance of A and D, respectively. In addition, it is interesting to highlight that A and D were reported in K-OLP without pain, as well as severe pain being reported also in K-OLP without A and D. Instead, A and D were strictly interconnected in patients with K-OLP as shown both by the dependence and multiple regression analyses. Indeed, D could explain 55.5% of the variance of A and A contributed to 56.2% of the variance of D. In contrast, the other variables considered together could increase the R2 value by only 4.3% and 2.5% for A and D, respectively.

Therefore, from this analysis, it is possible to suppose that the symptomatology was not consistently interconnected with A and D, as shown in a previous study where the worsening of symptoms was directly associated with an increase in psychological distress. [1] Instead, it is potentially related to peripheral neuropathy, as suggested in our previous research [6] in which the subjective perception of pain was predominantly related to the extension of the disease, independently of the clinical form of OLP. Moreover, in line with previous studies [26, 32], the A and D levels may not be significantly correlated with the severity of OLP, considering that all the patients were affected by keratotic lesions.

In the current study, the disease onset was at about 4.5 years. Therefore, it is not possible to exclude the possibility that the fear of cancerization of the disease has had an impact on susceptible patients in terms of the development of psychological distress over time. Indeed, in line with previous studies [31, 33], in this sample the prevalence of A and D was higher in women, generally considered more vulnerable to stress and more prone to develop psychiatric diseases, especially during perimenopausal endocrine changes. In addition, as suggested by the study of Mehdipor et al. [34], patients suffering from OLP have a greater tendency to experience anger, repressed and not expressed, compared to healthy subjects. Therefore, it may be possible to speculate that the continuous failure to express individual emotions, over time, may predispose subjects to a more serious psychological impairment [35].

The similarity in the biological pathways between A, D, and OLP may be explained by the involvement of the serotonergic system, which could be implicated also in the pathogenesis of OLP, as described by Kurmus et al. [13] Another possible explanation may involve the bidirectional connection between the immune system and central nervous system, in which mood disorders may influence the clinical expression of OLP by working on the functions of the immune system, which in turn may cause or aggravate neuropsychiatric diseases through the production of proinflammatory cytokines, such as tumor necrosis factor-alpha (TNF-α), interleukin-1 (IL-1), interleukin-6 (IL-6), interleukin-8 (IL-8), interleukin-10 (IL-10) and interleukin 17 (IL-17) [36–39].

Interestingly, recent researches have suggested the potential role of vitamin D in reducing the expression of some pro-inflammatory cytokines [40]. Specifically, vitamin D deficiency has been correlated to higher serum levels of IL-17 and IL-6 in OLP patients, especially in the symptomatic subset [41]. In addition, the local inflammatory response in the oral mucosa towards an unknown antigen may be responsible for the peripheral neuropathy, independently of the clinical form of OLP, causing in time pain and additional symptoms [42].

Moreover, recent studies have suggested a possible role of the imbalance of the oral microbiota and host response in the development of neurodegenerative and immune diseases [43, 44]. Therefore, the oral dysbiosis may be implicated in the onset of disease and in the pathogenesis of mood disorders by acting on the brain-gut axis [45]. Indeed, although the dysbiosis in the oral microbiota is more remarkable in non-keratotic OLP, it is found also in K-OLP patients and not only may be associated with a progression of this subtype towards an erosive form but is also in accordance with the high prevalence of A and D found in these subjects [46, 47].

The results of this study have confirmed that patients with K-OLP may suffer from A and D and a complex symptomatology that may potentially influence the clinical course and the evolution of the disease. Therefore, dentists, frequently consulted by such patients initially, must be aware of these comorbidities and should carefully and routinely evaluate emotional disorders, pain/burning, and additional symptoms in the assessment of all subtypes of OLP, at the first diagnosis and during follow-up. The evaluation of the psychological profile of the patient is complex in a dental setting and requires a learning curve on the part of clinicians since the majority of K-OLP patients are unaware that they are suffering from A or D and have never been specifically examined for mood disorders.

Further prospective studies using a structured clinical psychiatric interview should be carried out to assess the specific prevalence of A, D, and others psychiatric comorbidities in order to confirm our data and to better understand the cause-effect role between mood disorders and K-OLP.

### Limitations

The results of the present study should be interpreted in light of certain limitations. Indeed, due to the cross-sectional nature of the study, it is not possible to deduce any cause-effect relationship between mood disorders (A and D) and OLP, although their strong association is suggested.

Moreover, the recruitment of the participants was undertaken in tertiary referral Oral Medicine Units, with the result that potential confounding factors may have been introduced due to the heterogeneity of the different centers. Finally, the differential diagnosis between OLP and oral lichenoid drug reactions (OLDR), which also may appear at any time even years after the drug administration, was not feasible, as at the present there are no available diagnostic tests for OLDR [4].

## Conclusions

In this large multicentric Italian study, the prevalence of A and D in relation to K-OLP was significantly higher in comparison to the control group, suggesting a strong association between A, D, and K-OLP.

Moreover, almost 60% of K-OLP patients reported oral pain/burning and additional symptoms, also in sites without any lesions. As expected, patients suffering from higher levels of A and D, also reported higher scores of pain. Despite this positive correlation, A and D have been interestingly reported in relation to K-OLP without pain, as well as severe pain being reported also in K-OLP without A and D, suggesting that the oral discomfort may be predominantly related to the peripheral neuropathy, independently of the clinical form of the disease.

These findings may suggest that, although the neurological pathways of pain modulation and mood disorders (A and D) are similar and to some extent overlap and despite the well-established link between OLP, A, and D, there are probably other separate pathogenetic mechanisms implicated in pain perception and in the development of mood disorders which should be further elucidated.

In conclusion, any improvement in the psychological status of K-OLP patients, through appropriate treatment, may prevent the progression of the lesions and reduce the associated symptoms, thereby contributing to promote the patient’s recovery and improve the prognosis.

## Supplementary Information

Below is the link to the electronic supplementary material.

Supplementary file1 (DOCX 31 KB)

## Data Availability

The data that support the findings of this study is available from the corresponding author upon reasonable request.
